# A new approach for severe aortic regurgitation in porcelain aorta with sutureless Perceval valve: A case report

**DOI:** 10.1016/j.ijscr.2019.04.044

**Published:** 2019-05-13

**Authors:** Gian Luca Martinelli, Attilio Cotroneo, Edmond Stelian, Diana Benea, Marco Diena

**Affiliations:** a CardioVascular Dept., Clinica San Gaudenzio - Gruppo Policlinico di Monza, 3, Via Bottini, Novara, 28100, Italy; b Cardiac Anesthesiology Dept., Clinica San Gaudenzio - Gruppo Policlinico di Monza, 3 Via Bottini, Novara, 28100, Italy; c Cardiology Dept., Clinica San Gaudenzio - Gruppo Policlinico di Monza, 3,Via Bottini, Novara, 28100, Italy

**Keywords:** Aortic valve replacement, Aortic regurgitation, Porcelain aorta, Transcatheter aortic valve implantation, Sutureless valve

## Abstract

**Introduction:**

The association of pure aortic regurgitation and porcelain aorta represents a challenging situation. In the Transcatheter Aortic Valve Implantation (TAVI) era, porcelain aorta (PA) becomes an additional risk for patient treatment and sometimes serves as the primary indication for the TAVI approach, even in low-risk patients. Devices currently on the market are not yet validated for the treatment of pure aortic regurgitation (AR) in PA and mid/long-term results are still not available. Furthermore, small calcified sinotubular junction and the association of small Valsalva sinus with low origin of coronaries ostia represent a relative contraindication of TAVI.

**Presentation of case:**

We report a case of severe symptomatic AR associated with a PA in a patient successfully treated with a sutureless Perceval valve.

**Discussion:**

The sutureless Perceval valve may represent an excellent option. This valve requires less manipulation of the ascending aorta and no manipulation of the aortic annulus except for the aortic valve leaflets removal. Furthermore, it can be implanted also in a small and calcified sino-tubular junction because the valve is collapsible before the implant.

**Conclusion:**

The present case represents a proof that self-expandable cardiac valve technology can be employed to treat, either by surgery or by catheter, selected cases of AR. We have observed an excellent mid term result with no paravalvular leak at 2 years.

## Highlights

• Pure AR associated with porcelain aorta can be treated with the sutureless Perceval valve.
• The sutureless Perceval valve does not at all require sutures for fixation.
• The nitinol stent allows excellent fixation even in an elliptic calcified annulus.
• Self-expandable aortic valves are effective in the treatment of pure AR.

## 1 Introduction

In the past ten years, several new technologies and devices became available to treat aortic valve diseases. In the hands of cardiac surgeons and interventional cardiologists, they offer many alternatives in order to provide each case with the best solution. Guidelines summarize and evaluate available evidence, aiming to assist health professionals in selecting the best management strategies for each individual patient. Their recommendations facilitate decision-making. However, it is up to the health care professional to make the final decision concerning an individual treatment. Furthermore, due to the lack of evidence-based data in the field of cardiac valve disease, most recommendations are largely the result of expert consensus opinion. Deviations from these guidelines may be also appropriate in certain clinical circumstances, because in daily clinical practice, surgeons can deal with special situations.

The new European guidelines confirm that, in symptomatic patients with severe AR, surgery is recommended irrespective of the left ventricular ejection factor (LVEF) value, except for extreme conditions [1]. The use of risk scores have major limitations in practical use and they do not include major risk factors such as porcelain aorta. Furthermore, new devices such as surgical sutureless and transcatheter valves are not yet validated for the treatment of AR.

Porcelain aorta (PA) is an extensive calcification of the ascending aorta, which can be completely or near completely circumferential [[2], [3], [4]]. It is often associated with valvular and coronary calcifications, reflecting an underlying atherosclerotic process [5,6], with an increasing incidence in the elderly and in patients with coronary artery disease (CAD) or aortic stenosis (AS) [7,8]. The prevalence of PA was found to be 7.5% in patients evaluated for AS (8) and between 5% and 33% in the patients undergoing TAVI [9].

PA is associated with increased morbidity and mortality, especially as a result of higher perioperative stroke risk [10,11].

Amorim et al. [12] proposed classification of PA into type I, if circumferential calcification is present in the ascending aorta independently of further extension, and type II, if circumferential calcification is localized only in the aortic arch or descending aorta. Type I PA was further subdivided into type IA when there is no possibility to clamp the aorta during cardiac surgery and type IB when clamping is possible, but at increased risk.

We report a case of a patient presenting severe symptomatic AR, associated with a diagnosis of porcelain aorta, successfully treated with a sutureless Perceval valve. The work is in line with the Scare criteria (13).

## 2 Case report

**Fig. 1:**
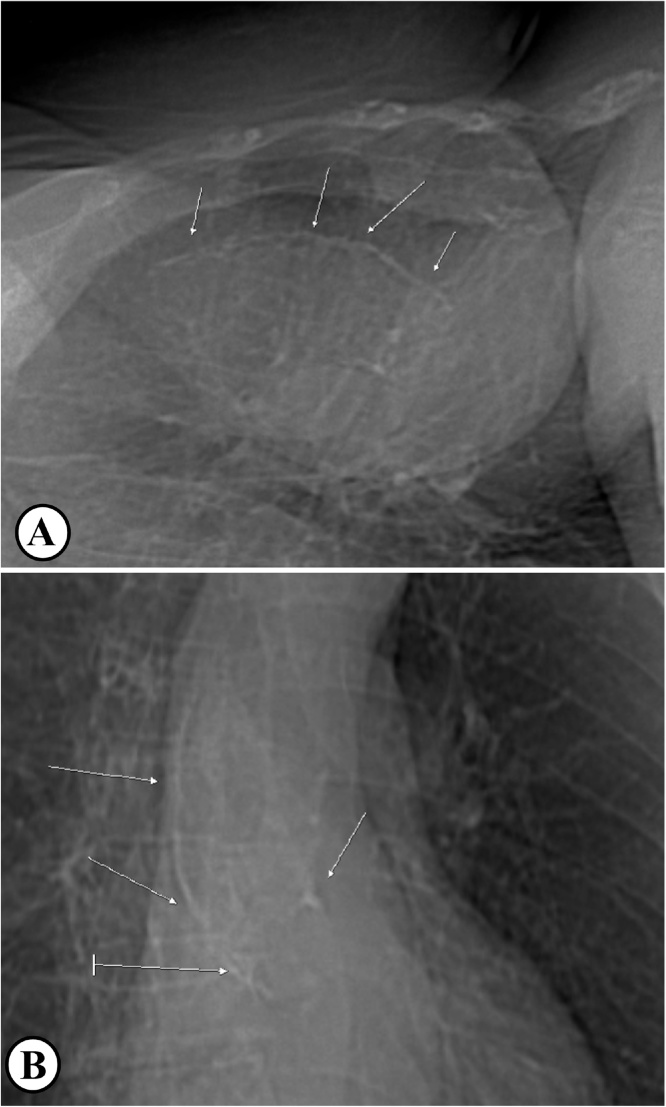
Chest X-Ray.

**Fig. 2:**
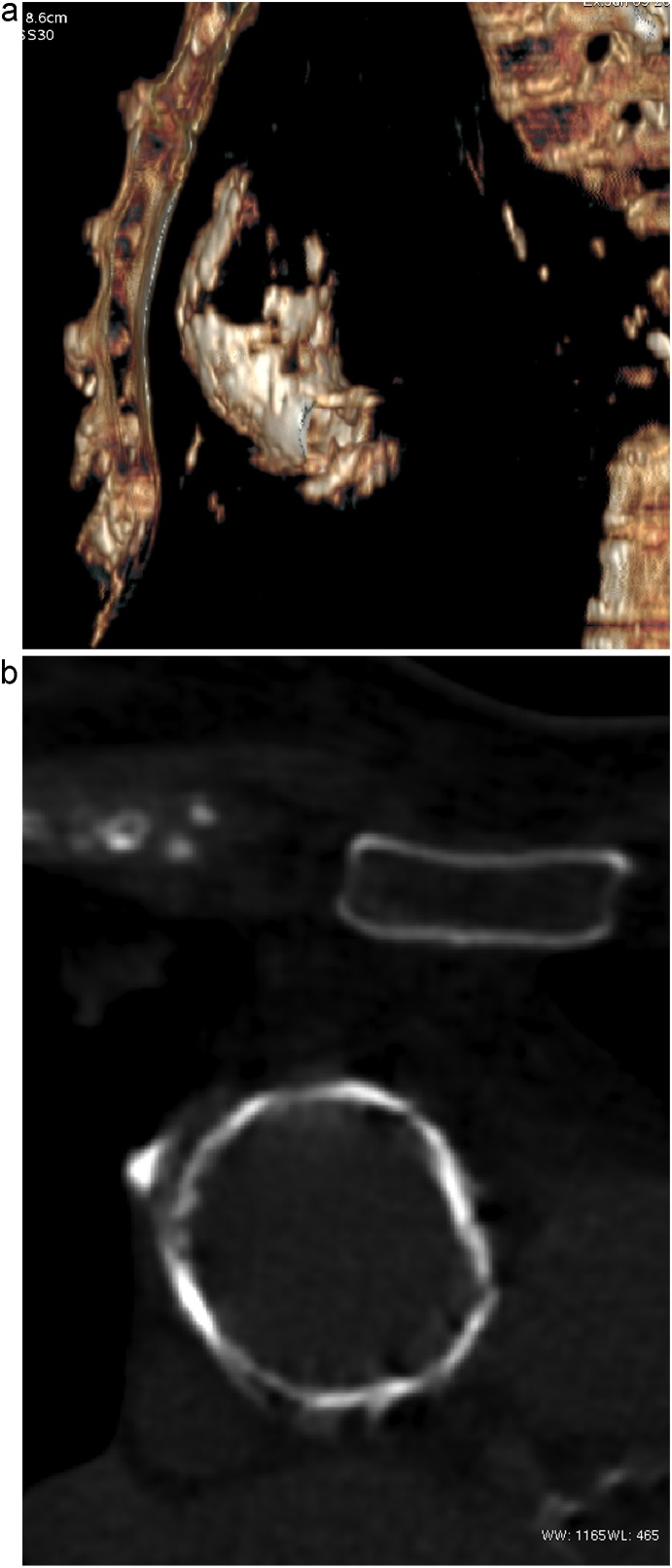
Computed tomography of the thorax that confirms a porcelain aorta: 2a scan volume rendering reconstruction, 2b: cross sectional view of contrast enhanced CT.

A woman, 70 years old, with dyspnoea (New York Heart Association scale, NYHA class III), referred for severe aortic regurgitation. At the transthoracic echocardiography (TTE) aortic valve presented with an annulus diameter of 23 mm, a mean gradient of 7 mmHg and a severe AR with a pressure half time (PHT) inferior at 300 msec. The effective regurgitant orifice was 0,3 cm^2^ and the regurgitant volume was 65 ml. The AR was associated with a left ventricular dilatation with a left ventricle end diastolic volume (LVEDV) of 160 ml, left ventricle end diastolic (LVEDD) and systolic (LVESD) diameters of 57 mm and 41 mm, and a normal ejection fraction (60%). The patient was previously treated for a myocardial infarction with drug eluting stents in the right coronary artery (RCA) and in the circumflex artery, and with another drug eluting stent in RCA for late in-stent restenosis. She had a stenosis of 55% of the left internal carotid artery and severe peripheral vascular disease. Preoperative Chest X-ray (Fig. 1) and computed tomography (CT) of the thorax (Fig. 2) confirmed the presence of a PA. In particular, CT showed heavy and diffuse calcifications involving all the aortic annulus and the aortic root and numerous large spots of calcium from the sinotubular junction to the upper portion of the ascending aorta. These findings allowed for a diagnosis of type IB PA.

The patient’s expected operative risk, calculated according to the logistic European System for Cardiac Operative Risk Evaluation (EuroSCORE II), was 2,24%.

We evaluated the available options and decided for an aortic valve replacement (AVR) with a sutureless Perceval valve (LivaNova, Saluggia, Italy) implanted in full sternotomy.

The ascending aorta was cannulated in a restricted safe area, previously identified on CT images, by Seldinger technique (EOPAtm, Medtronic-Inc, Minneapolis-USA), then cardiopulmonary bypass was instituted, and the aorta was clamped in a non-calcified ascending aorta segment. Myocardial protection was obtained with a single dose of warm blood cardioplegia delivered selectively in the two coronary ostia. Aortic valve leaflets were removed, and a Perceval valve (M-size) was implanted without using the 3 guiding sutures, because of huge and totally diffusing calcifications involving the aortic annulus and the aortic root. To correctly position the valve in the native annulus, the surgeon found the solution of carefully measuring the distance between the nadir of each cusp and the aortotomy. Closure of the aortotomy was reinforced with two layers of autologous pericardium. The cardiac arrest time was 45 min, the cardio-pulmonary bypass (CPB) time was 59 min. Intraoperative transoesophageal echocardiographic assessment showed no paravalvular leakages (PVL). The patient was extubated 7 h later in the intensive care unit. The correct position of the valve was confirmed by the postoperative TTE.

In the postoperative period we noticed an atrial fibrillation with left bundle branch block and an asystole of 7 s; those findings lead us to decide to implant a bicameral pacemaker. The patient was discharged on the 7th postoperative day.

**Fig. 3:**
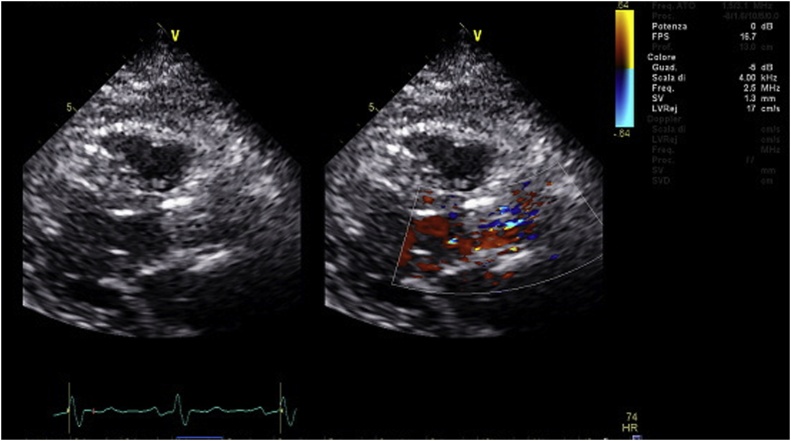
Post operative echocardiography shows correct position of the Perceval sutureless valve with no para-valvular leaks.

At 2 years follow-up the patient was in NYHA class I and TTE showed no PVL (Fig. 3), a mean gradient of 11 mmHg, LVEDV, LVESD and LVEDD of 85 ml, 32 mm and 43 mm, and left ventricular ejection fraction was 66%.

## 3 Discussion

In the TAVI era, PA became an additional factor for patient selection, and sometimes serves as the primary indication for the TAVR approach even in low-risk patients [4].

An exact diagnosis of PA requires an accurate analysis in order to identify the extension of the aortic calcifications.

Fluoroscopy use during coronary angiography can also show diffuse, generalized calcifications of the ascending aorta, suggesting the diagnosis of PA; however this is not representing an accurate modality for the assessment of the aortic calcifications extent. Occasionally, a chest X-ray might reveal calcific outline of the ascending aorta or the aortic arch (Fig. 1).

Indeed, Multislice (spiral) CT is an effective, non-invasive technique for pre-procedural cardiac, coronary, and aortic calcification imaging [13]. Its use for the assessment of PA during the preoperative patients’ workup, may help elucidate the true incidence, planning the clinical implications of PA in case of chest x-ray or echocardiographic suspicions (Fig. 2) [14].

Aortic valve surgical alternatives in patients with PA include low aortotomy in the ascending aorta, if a non-calcified area is present, or replacement of the ascending aorta [9]. The endoaortic balloon clamp, used as an alternative to the external application of a classic aortic cross-clamp in mitral valve surgery and coronary artery bypass grafting, cannot be considered in AVR surgery, according to labelling, and mainly in this case due to the high risk of aortic dissection/rupture. If cross clamp is impossible, the only option is circulatory arrest. Although most of the procedures can be performed in less than 20 min of circulatory arrest, there may be some reluctance to perform deep hypothermic circulatory arrest in patients with advanced age and significant comorbidities [15].

When ascending aorta clamp is feasible, the sutureless Perceval valve may represent an excellent option [[16], [17], [18]]. This valve requires less manipulation of the ascending aorta and no manipulation of the aortic annulus except for the aortic valve leaflets removal. Furthermore, it can be implanted also in small and calcified sino-tubular junction thanks to the fact that the valve is collapsible before the implant.

In the reported case, the planning of a conventional AVR was abandoned due to the diagnosis of a porcelain ascending aorta, and for the need of suturing the prosthesis in the calcified aortic annulus. The transcatheter solution was also evaluated but excluded due to patient age and several technical reasons:

• only trans-apical access was feasible (peripheral vascular disease and PA with involvement of both subclavian arteries);
• presence of a calcified and small sino-tubular junction (19 mm) that did not allow a balloon-expandable prosthesis;
• small Valsalva sinus associated with low origin of coronary arteries.

For all these reasons, the sutureless Perceval aortic valve was our choice. Other advantages of Perceval are:

• it is a real sutureless valve, so it does not require sutures for fixation at all;
• the nitinol stent allows an excellent fixation and stability even in elliptic calcified annulus;
• the CPB time is shortened.

## 4 Conclusion

The association of pure AR and PA represents a challenging situation. An alternative to classical surgical approach with hypothermic circulatory arrest can be a TAVI implantation, even if those devices are not validated in pure AR. This case report proves that self-expandable cardiac valve technology can be employed to treat, either by surgery or by catheter, selected cases of AR.

## Conflicts of interest

All the authors do not have any financial and personal relationships with other people or organisations that could inappropriately influence (bias) their work.

## Sources of funding

No fund for this research. Nothing to declare.

## Ethical approval

Ethical approval not necessary for Italian low for this case report. The bioprosthesis, Sorin Perceval suturless valve (LivaNova Sorin), used in this case has received,in 2012, CE mark approval for adult age indication allowing treatment of a wider spectrum of clinical indication for aortic valve replacement.

## Consent

Written informed consent was obtained from the patient for publication of this case report and accompanying images. A copy of the written consent is available for review by the Editor-in-Chief of this journal on request.

## Author contribution

Gian Luca Martinelli: Writing-Reviewing-Editing.

Attilio Cotroneo: Data curation-Original draft preparation.

Emond Stellian: Investigation.

Diana Benea: Supervision.

Marco Diena: Validation.

## Registration of research studies

Nothing to declare.

## Guarantor

Gian Luca Martinelli, MD.

## Provenance and peer review

Not commissioned, externally peer-reviewed
